# Characterization of microRNA expression profiles in normal human tissues

**DOI:** 10.1186/1471-2164-8-166

**Published:** 2007-06-12

**Authors:** Yu Liang, Dana Ridzon, Linda Wong, Caifu Chen

**Affiliations:** 1 Molecular and Cell Biology-R&D, Applied Biosystems, Foster City, CA 94404, USA

## Abstract

**Background:**

Measuring the quantity of miRNAs in tissues of different physiological and pathological conditions is an important first step to investigate the functions of miRNAs. Matched samples from normal state can provide essential baseline references to analyze the variation of miRNA abundance.

**Results:**

We provided expression data of 345 miRNAs in 40 normal human tissues, which identified universally expressed miRNAs, and several groups of miRNAs expressed exclusively or preferentially in certain tissue types. Many miRNAs with co-regulated expression patterns are located within the same genomic clusters, and candidate transcriptional factors that control the pattern of their expression may be identified by a comparative genomic strategy. Hierarchical clustering of normal tissues by their miRNA expression profiles basically followed the structure, anatomical locations, and physiological functions of the organs, suggesting that functions of a miRNA could be appreciated by linking to the biologies of the tissues in which it is uniquely expressed. Many predicted target genes of miRNAs that had specific reduced expression in brain and peripheral blood mononuclear cells are required for embryonic development of the nervous and hematopoietic systems based on database search.

**Conclusion:**

We presented a global view of tissue distribution of miRNAs in relation to their chromosomal locations and genomic structures. We also described evidence from the *cis*-regulatory elements and the predicted target genes of miRNAs to support their tissue-specific functional roles to regulate the physiologies of the normal tissues in which they are expressed.

## Background

MicroRNAs (miRNAs) belong to a family of small non-coding RNAs (18~22 nucleotides) that interact with their target coding mRNAs to inhibit translation by either degradation of the mRNAs, or blocking translation without degrading the targets [1]. Significant conservation of individual miRNAs across different species suggests their functional importance. It has been shown in several animal models that miRNAs participate in determination of cell fate, pattern formation in embryonic development, and in control of cell proliferation, cell differentiation, and cell death [2]. Therefore, it is reasonable to speculate that miRNAs are also involved in human diseases such as cancers [3]. Several groups of miRNAs have been identified to regulate the expression of tumor-associated genes [4], while others seem to hold prognostic value in predicting the survival of cancer patients [5].

Chromosomal location and genomic distribution of a miRNA gene are important determinants of its expression from at least three perspectives. First, about 80% of miRNA genes are located within introns of defined transcription units [6], and their expression is frequently correlated with the expression profiles of their host genes [7,8]. Second, many miRNA genes are distributed as clusters, and a microarray expression profiling of 175 miRNAs in 24 human tissues showed that proximally paired miRNA genes at a distance up to 50 kb are generally co-expressed [8]. The best example may be the four miRNA genes (miR-196b, miR-10a, miR-196a-2, and miR-10b) that are embedded in the Hox gene clusters (*Hox A*, *Hox B*, *Hox C*, and *Hox D*, respectively). By histochemical staining and *in situ *hybridization, expression patterns of miR-10a and Hoxb4 mRNA are very similar, suggesting that they share regulatory control of transcription [9]. Lastly, miRNA genes are frequently located at fragile sites, as well as in regions of loss of heterozygosity, regions of amplification, or common breakpoint regions [10]. Expression of miRNA genes within the regions afflicted by chromosomal aberration, a hallmark characteristic of neoplastic cells, could also be directly affected. For example, miR-15a and miR-16-1 are located at a frequently deleted site in most of the B cell chronic lymphocytic leukemia patients [11], and induce apoptosis in a leukemia cell line model [12].

Most expression profiling of miRNAs in normal human tissues has been explored in a rather small collection of tissues or miRNAs, in which some of them were restricted by time-consuming and laborious strategies such as Northern blotting or cloning [13]. One report used a bead-based detection platform to profile expression of 217 miRNAs in a broad spectrum of normal human tissues, but low sensitivity and specificity make the results problematic for miRNAs that are less abundant [14]. Microarrays have the advantage of high throughput and was used for profiling expression of miRNAs [8,15,16], but they have the same concern of sensitivity, and it might be difficult for them to differentiate closely related miRNAs in sequences.

Sensitivity is always a major obstacle to examine tissue-specific expression patterns of miRNAs with low abundance. A new type of real time reverse transcription (RT)-PCR-based miRNA assays were recently developed that have better sensitivity and specificity compared to bead- and microarray-based technologies [17]. We used these assays to examine global profiles of distribution and expression of 345 unique miRNAs in 40 normal human tissues, so we could identify tissue-specific miRNAs that provide foundations to pursue diagnostic and therapeutic targets as well as molecular mechanisms underlying the phenotypic diversity of different tissues, and present universal baselines for investigating variations in miRNA expression under physiological or pathological conditions. Our data were also combined with public datasets to systematically analyze the association between genomic locations of miRNAs and their expression, and the correlation of expression between miRNAs and their predicted target genes.

## Results

### Hierarchical clustering of normal human tissues is mainly based on their anatomical locations and physiological functions using the miRNA expression profiles

**Figure 1:**
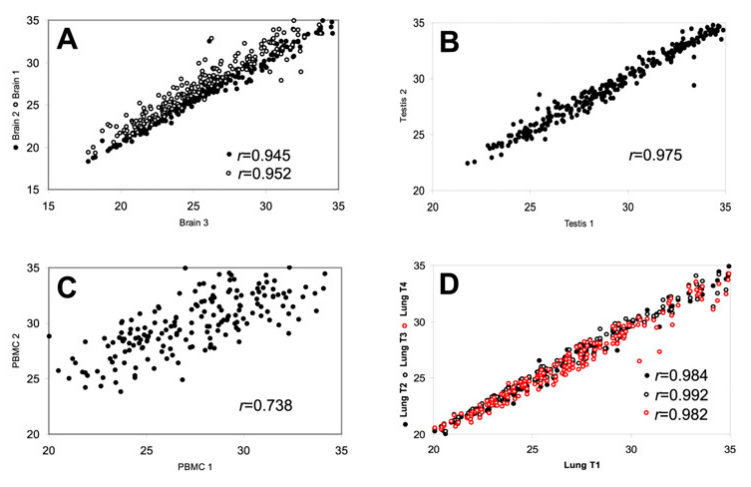
Scatter plots demonstrate the reproducibility and repeatability of the TaqMan^® ^miRNA assays. We examined the miRNA expression in three brain (A), two testes (B), and two PBMC (C) specimens. Assays that had C_T _values > 35 were removed from the analysis and correlation of the data were evaluated by Spearman test. Expression of miRNAs was also examined in the same lung specimen at four different time points (T1 to T4) within a five-month time frame to show the repeatability of the data (D).

To assess the reproducibility of the TaqMan^® ^MicroRNA Assays, we characterized the expression of miRNAs in three brain, two testes, and two peripheral blood mononuclear cells (PBMC) specimens. We found a high concordance between the three brain specimens (*r *= 0.945 and 0.952, respectively, Figure 1A), between the two testes (*r *= 0.975, Figure 1B), and to a lesser extent between the two PBMC samples (*r *= 0.738, Figure 1C). To demonstrate the repeatability of the data during the course of our studies, we examined the expression of miRNAs in the same lung tissue at four different time points within a five-month time frame, and the abundance of miRNAs we measured were highly consistent at all times (*r *> 0.98, Figure 1D).

**Figure 2:**
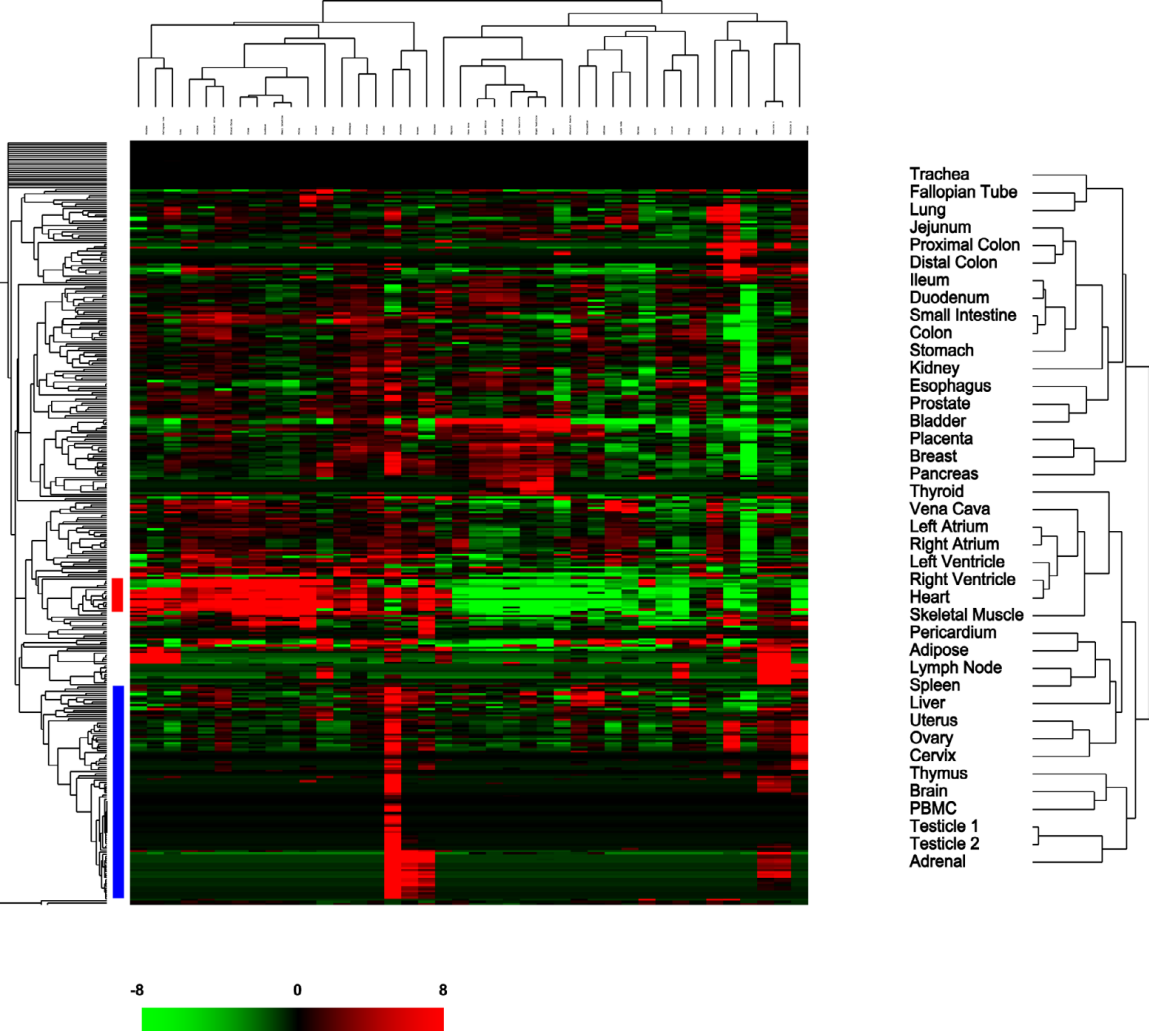
Unsupervised hierarchical clustering of the normal human tissues based on the variation of miRNA expression correlates with the anatomical locations and physiological functions of the tissues. Normalized C_T _for each assay was transformed into ΔC_T _against the average C_T _of all assays examined and clustered after mean-centering the data for each miRNA but no centering was done for the tissues. A detailed view of the clustering patterns of normal tissues is on the right. The blue bar on the left side of the heat map represented the group of miRNAs primarily expressed in placenta, and the red bar indicated the miRNAs with significant increased expression in epithelial tissues including the gastrointestinal organs. A pseudocolor scale bar represented the fold change relative to the mean of the data for each miRNA.

The expression of 345 human miRNAs was quantitated in a spectrum of 40 normal human tissues that included specimens derived from brain, muscle, circulatory, respiratory, lymphoid, gastrointestinal, urinary, reproductive, and endocrine systems (see Additional file 1). We employed an unsupervised hierarchical clustering based on the variation of expression for each miRNA across the specimens examined to explore the correlation between different tissue types. In general, normal human tissues derived from similar anatomical locations or with related physiological functions were primarily clustered together (Figure 2). For example, tissues derived from different parts of heart (atrium versus ventricle) were clustered with skeletal muscle. Tissues from the gastrointestinal system (stomach, small intestine, and colon), lymphoid tissues (spleen and lymph node), female reproductive organs (ovary, uterus, and cervix), and respiratory tissues (lung and trachea) were also together, respectively, as shown in Figure 2. This result recapitulated the previously published clustering patterns of normal tissues using mRNA expression profiles [18].

However, the clustering patterns among some tissue types by their mRNA and our miRNA expression profiles were quite different (see Additional file 2). Lung was clustered together with female reproductive organs and esophagus by mRNA expression profile, but miRNA expression profile of lung was only similar to that of Fallopian tube. Thyroid was similar to different parts of the heart in miRNA expression but not in mRNA expression. Liver was clustered together with the gastrointestinal organs and kidney by mRNA expression profiles but not when the miRNA expression profiles were used. Brain, PBMC, thymus, adrenal gland, and testes formed a unique cluster separate from the other tissues by their miRNA expression profiles, but such similarities in mRNA expression profiles between these five tissue types were only observed separately between brain and testes, and between thymus and PBMC [18].

Some groups of miRNAs demonstrated highly differential patterns of tissue distribution that were not seen in the mRNA profiles. For example, a prominent expression of a group of miRNAs (miR-141, miR-200 family, miR-429, miR-375, and miR-31) mainly in epithelial tissues, such as lung, breast, and the gastrointestinal organs (*r *= 0.72, Figure 2), contributed to separate all the normal tissues examined into two parts. A neighboring group of miRNAs (miR-192, miR-194, and miR-215) shared similar expression patterns but particularly in the gastrointestinal organs (*r *= 0.912, Figure 2). A large number (~100) of miRNAs had pronounced expression in placenta compared to most of the other tissues.

### Localization in the same genomic cluster is the most recognizable feature for miRNAs that have correlated abundance and expression patterns among tissues

**Figure 3:**
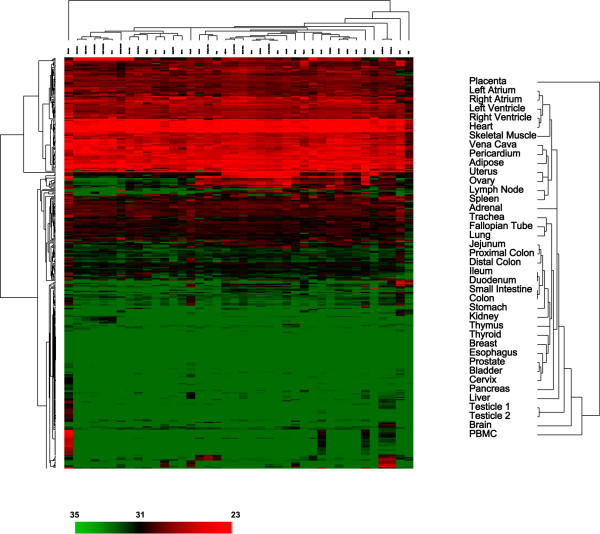
Unsupervised hierarchical clustering of normal human tissues based on the variation of miRNA abundance demonstrates similar patterns as shown in Figure 2. Normalized C_T _for each assay was transformed into ΔC_T _against the average C_T _of all assays examined and clustered without centering the data. A pseudocolor scale outlines the C_T _values represented in the heat map. A detailed view of the clustering patterns of normal tissues is on the right.

Centered expression data for each miRNA as shown in Figure 2 describes the "pattern" of expression in tissues without regarding the abundance of that miRNA, but identification of tissue-specific markers or potential diagnostic/therapeutic targets requires unmodified quantitative measurements from the TaqMan^® ^assays. Our uncentered expression data revealed both miRNAs universally expressed in all tissues as well as those differentially expressed among samples (Figure 3 and see Additional file 3). Classification of the normal tissues using the uncentered data maintained some of the patterns observed in Figure 2, for example, the gastrointestinal organs, different parts of the heart, lymphoid tissues, lung and trachea, and female reproductive organs. However, placenta and PBMC were separate from the rest of tissues in the hierarchical clustering because their miRNA expression levels were distinctive from those of other tissues (Figure 3).

**Figure 4:**
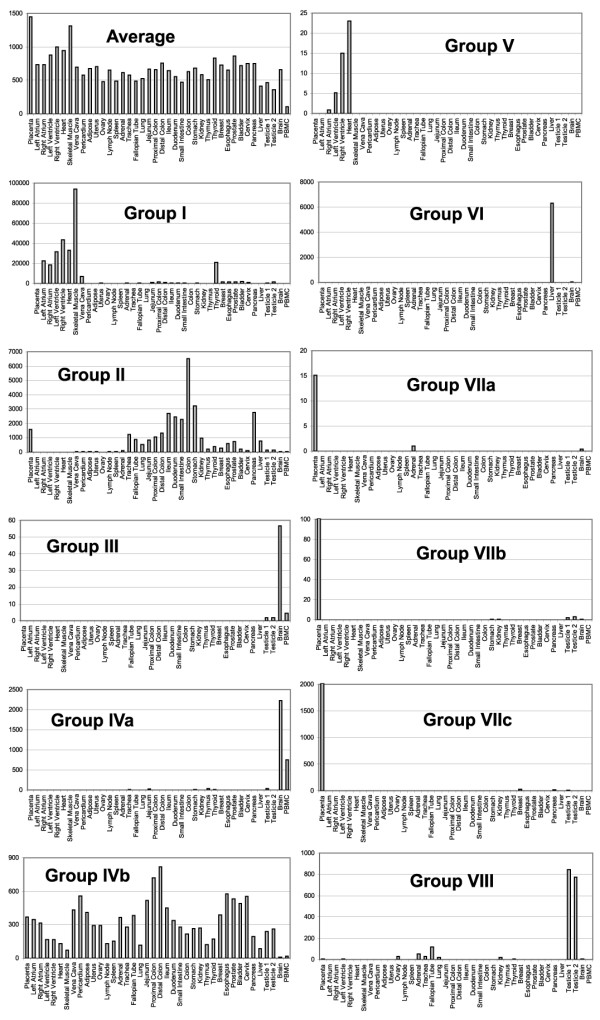
The abundance of miRNAs in all tissues represented by the estimated average copy numbers of all miRNAs examined, as well as by the average copy numbers of miRNAs in each of the eight most differentially expressed groups. Y-axis is the estimated copy number per cell (assuming 30 pg of total RNA in each cell), and the order of normal tissues at the X-axis is arranged by the clustering patterns shown in the Figure 3.

Estimated average copy numbers converted from C_T _values for all miRNAs examined in placenta and PBMC were approximately 1,500 and 100 copies, respectively (Figure 4, and see Additional file 4 for complete copy number data). Estimated average miRNA copy numbers from these two tissue types were significantly different from those of the rest of tissues (p = 0.0013 for placenta and p = 4 × 10^-71 ^for PBMC by Student's *t *test).

Three criteria were used to define universally expressed miRNAs if their (1) average C_T _values in all tissues were less than 30, (2) standard deviations of C_T _values in all tissues were less than 0.8, and (3) their maximal and minimal C_T _values in tissues differed by less than 4 (yellow highlighted miRNAs in the Additional file 1). Since the 15 miRNAs identified, which include the 4 miRNAs used to normalize our data (see Methods), showed rather consistent expression levels in the extensive list of tissues we surveyed, they would be strong candidates for normalizing miRNA expression should the types of normal human tissues beyond our list be examined. One of the 15 miRNAs, miR-16, has been found abundantly expressed in all tissues and was used as a control in several systems including animal models [19].

**Figure 5:**
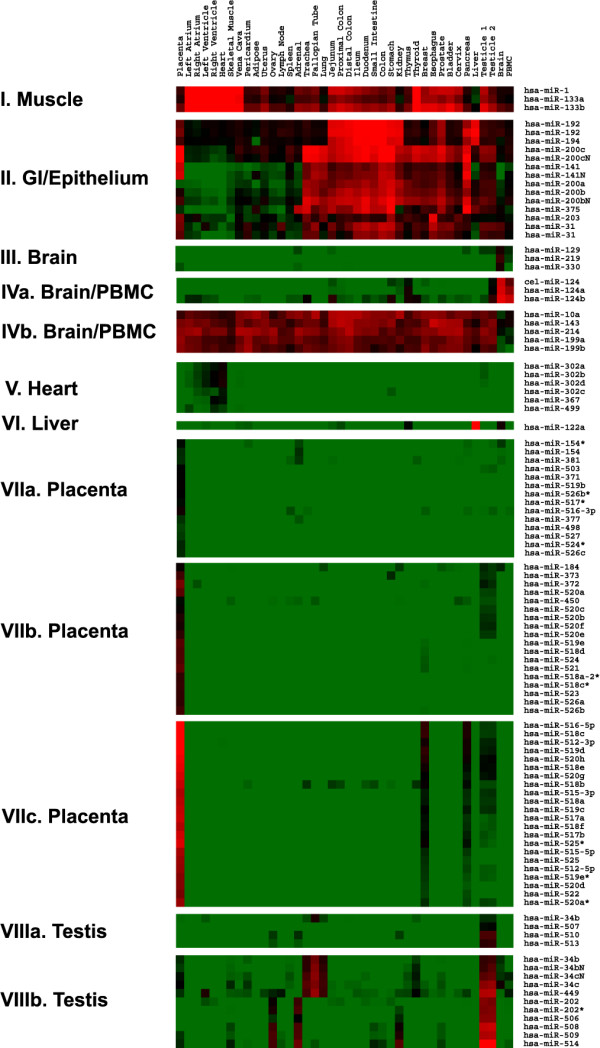
An enlarge view of the eight groups of most differentially expressed miRNAs. The pseudocolor scale is the same as that in Figure 3.

Because we did not have replicate samples for most tissue types, it is not sensible to use class prediction/marker discovery programs to identify tissue-specific miRNA markers or differentially expressed miRNAs. Instead, we used the following four criteria to select eight miRNA groups (Figure 5). First, correlation coefficient (*r*) of the miRNA expression patterns in the group was larger than 0.9. Second, the miRNAs were differentially expressed in tissues from similar anatomical locations and/or with similar physiological functions as the clustering shown by Figures 3 and 5. Third, the miRNAs were preferentially expressed in one tissue type or few organ sites that do not appear to have obvious physiological link. Lastly, the miRNAs in each group that are located at the same genomic cluster were statistically overrepresented (by Chi-square test, see below).

Group I (*r *= 0.937) that contains miR-1 and miR-133a/b showed highest expression in different parts of the heart and skeletal muscle as well as in vena cava, and in, unexpectedly, thyroid. This expression pattern is consistent with previous observations in their localization and functional analysis [8,20]. However, it was not previously appreciated that much lower expression of miR-1 and miR-133a/b was seen in some non-heart, non-skeletal muscle tissues. These are "hollow" organs composed of smooth muscle-containing wall, such as the gastrointestinal system, suggesting that expression of miR-1 and miR-133a/b might mark some features shared by different muscle types (i.e., skeletal, cardiac, and smooth). The group II shown in Figure 5 included two subgroups with similar expression patterns, in that miRNAs in the first subgroup (miR-192 and miR-194, *r *= 0.988) had rather focused expression in the gastrointestinal organs as well as in kidney. The second subgroup was included despite slightly lower correlation coefficient (*r *= 0.832) because it appeared to be expressed mainly in organs lined with epithelium, such as the gastrointestinal system, urinary system, and lung, but not heart, muscle, lymphoid tissues, liver, brain, and PBMC. Group III (*r *= 0.995) and group IVa (*r *= 0.96) miRNAs had preferential expression in brain and PBMC, whereas concurrent lack of expression of the group IVb miRNAs (*r *= 0.96) was seen in brain and PBMC. Very low but specific expression of the members of the mir-302 family and miR-367 and miR-499 (group V, *r *= 0.995) was detected in different parts of the heart. Expression of liver-specific miR-122a [21] was confirmed by our data, but we also saw very low copy number of this miRNA in brain (34 copies) and thymus (19 copies). There were three subgroups (group VIIa, *r *= 0.998; group VIIb, *r *= 0.992; group VIIc, *r *= 0.99) and two subgroups (group VIIIa, *r *= 0.983; group VIIIb, *r *= 0.909) of miRNAs that had preferential expression in placenta and testes, respectively, as they had minimal expression in most other tissues in each subgroup (Figure 5).

The miRNAs in some of the eight differentially expressed groups identified in Figures 5 seemed to be localized within the same genomic region, usually 1 to 5 kb apart from each other (see Table 1 for chromosomal locations). Two examples are the groups of miRNAs that had preferential expression in placenta and testes that are localized in two separate genomic clusters at chromosomes 19q13.42 and Xq27.3, respectively. We used Chi-square test to evaluate the statistical significance of the presence of genomically clustered miRNAs in each expression group based on the expected and observed frequency of clustered miRNAs. This testing was rather stringent because the clustering analysis on which the eight differentially expressed groups were based primarily measured the abundance of miRNAs; therefore, miRNAs that were expressed at different levels in tissues would not be clustered into the same expression group despite their similar expression patterns among the tissues examined. The best example is the three miRNAs (miR-381/154/377) in the group VIIa that are located within a cluster of at least 32 miRNAs at chromosome 14q32.31. The other miRNAs in the same genomic cluster were not in the group VIIa because of their variable abundance. When centered data were used, in which the expression pattern was primarily measured, all these miRNAs were clustered together (represented by blue bar in Figure 2). For this reason, genomically clustered miRNAs from all subgroups of the differentially expressed groups VII or VIII, were evaluated together. As summarized in Table 1, almost all miRNAs located in the same genomic clusters were overrepresented in the differentially expressed groups (in boxes, with significant p values).

**Table 1:** Genomic locations of miRNAs in the 8 differentially expressed groups.

	Ch	Start	End	Strand	p value*
**Group I**					
hsa-mir-133b	6	52121680	52121798	+	
hsa-mir-133a-1	18	17659657	17659744	-	
hsa-mir-1-2	18	17662963	17663047	-	1.77 × 10^-13^
hsa-mir-1-1	20	60561958	60562028	+	
hsa-mir-133a-2	20	60572564	60572665	+	
					
**Group II**					
hsa-mir-200b	1	1092347	1092441	+	
hsa-mir-200a	1	1093106	1093195	+	
hsa-mir-194-1	1	218358122	218358206	-	
hsa-mir-375	2	219574611	219574674	-	
hsa-mir-31	9	21502114	21502184	-	
hsa-mir-192	11	64415185	64415294	-	0.000039
hsa-mir-194-2	11	64415403	64415487	-	
hsa-mir-200c	12	6943123	6943190	+	
hsa-mir-141	12	6943521	6943615	+	
hsa-mir-203	14	103653495	103653604	+	
					
**Group III**					
hsa-mir-219-1	6	33283590	33283699	+	
hsa-mir-129-1	7	127635161	127635232	+	
hsa-mir-219-2	9	130194718	130194814	-	
hsa-mir-129-2	11	43559520	43559609	+	
hsa-mir-330	19	50834092	50834185	-	
					
**Group IVa**					
hsa-mir-124a-1	8	9798308	9798392	-	
hsa-mir-124a-2	8	65454260	65454368	+	
hsa-mir-124a-3	20	61280297	61280383	+	
					
**Group IVb**					
hsa-mir-214	1	170374561	170374670	-	0.000016
hsa-mir-199a-2	1	170380298	170380407	-	
hsa-mir-143	5	148788674	148788779	+	
hsa-mir-199b	9	130046821	130046930	-	
hsa-mir-10a	17	44012199	44012308	-	
hsa-mir-199a-1	19	10789102	10789172	-	
					
**Group V**					
hsa-mir-367	4	113788479	113788546	-	
hsa-mir-302d	4	113788609	113788676	-	
hsa-mir-302a	4	113788788	113788856	-	7.3 × 10^-12^
hsa-mir-302c	4	113788968	113789035	-	
hsa-mir-302b	4	113789090	113789162	-	
hsa-mir-499	20	33041840	33041961	+	
					
**Group VI**					
hsa-mir-122a	18	54269286	54269370	+	
					
**Group VIIa**					
hsa-mir-381	14	100582010	100582084	+	
hsa-mir-154	14	100595845	100595928	+	
hsa-mir-377	14	100598140	100598208	+	
hsa-mir-498	19	58869263	58869386	+	
hsa-mir-526b	19	58889459	58889541	+	
hsa-mir-519b	19	58890279	58890359	+	
hsa-mir-526a-1	19	58901318	58901402	+	
hsa-mir-524	19	58906068	58906154	+	
hsa-mir-517a	19	58907334	58907420	+	
hsa-mir-517b	19	58916142	58916208	+	
hsa-mir-516-3	19	58920508	58920592	+	0.026
hsa-mir-526a-2	19	58921988	58922052	+	
hsa-mir-516-4	19	58931911	58932000	+	
hsa-mir-517c	19	58936379	58936473	+	
hsa-mir-527	19	58949084	58949168	+	
hsa-mir-516-1	19	58951807	58951896	+	
hsa-mir-516-2	19	58956199	58956288	+	
hsa-mir-371	19	58982741	58982807	+	
hsa-mir-503	X	133508024	133508094	-	
					
**Group VIIb**					
hsa-mir-184	15	77289185	77289268	+	
hsa-mir-520e	19	58870777	58870863	+	
hsa-mir-519e	19	58875006	58875089	+	
hsa-mir-520f	19	58877225	58877311	+	
hsa-mir-520a	19	58885947	58886031	+	
hsa-mir-526b	19	58889459	58889541	+	
hsa-mir-523	19	58893451	58893537	+	
hsa-mir-520b	19	58896293	58896353	+	
hsa-mir-526a-1	19	58901318	58901402	+	
hsa-mir-520c	19	58902519	58902605	+	0.026
hsa-mir-518c	19	58903801	58903901	+	
hsa-mir-524	19	58906068	58906154	+	
hsa-mir-521-2	19	58911660	58911746	+	
hsa-mir-526a-2	19	58921988	58922052	+	
hsa-mir-518a-1	19	58926072	58926156	+	
hsa-mir-518d	19	58929943	58930029	+	
hsa-mir-518a-2	19	58934399	58934485	+	
hsa-mir-521-1	19	58943702	58943788	+	
hsa-mir-372	19	58982956	58983022	+	
hsa-mir-373	19	58983771	58983839	+	
hsa-mir-450-1	X	133502037	133502127	-	
hsa-mir-450-2	X	133502204	133502303	-	
					
**Group VIIc**					
hsa-mir-512-1	19	58861745	58861828	+	
hsa-mir-512-2	19	58864223	58864320	+	
hsa-mir-515-1	19	58874069	58874151	+	
hsa-mir-519e	19	58875006	58875089	+	
hsa-mir-515-2	19	58880075	58880157	+	
hsa-mir-519c	19	58881535	58881621	+	
hsa-mir-520a	19	58885947	58886031	+	
hsa-mir-525	19	58892599	58892683	+	
hsa-mir-518f	19	58895081	58895167	+	
hsa-mir-518b	19	58897803	58897885	+	
hsa-mir-518c	19	58903801	58903901	+	
hsa-mir-517a	19	58907334	58907420	+	
hsa-mir-519d	19	58908413	58908500	+	0.026
hsa-mir-520d	19	58915162	58915248	+	
hsa-mir-517b	19	58916142	58916208	+	
hsa-mir-520g	19	58917232	58917321	+	
hsa-mir-516-3	19	58920508	58920592	+	
hsa-mir-518e	19	58924904	58924991	+	
hsa-mir-518a-1	19	58926072	58926156	+	
hsa-mir-516-4	19	58931911	58932000	+	
hsa-mir-518a-2	19	58934399	58934485	+	
hsa-mir-520h	19	58937578	58937665	+	
hsa-mir-522	19	58946277	58946363	+	
hsa-mir-516-1	19	58951807	58951896	+	
hsa-mir-516-2	19	58956199	58956288	+	
					
**Group VIIIa**					
hsa-mir-34b	11	110888873	110888956	+	
hsa-mir-513-1	X	146102673	146102801	-	
hsa-mir-513-2	X	146115036	146115162	-	0.00001
hsa-mir-507	X	146120194	146120287	-	
hsa-mir-510	X	146161545	146161618	-	
					
**Group VIIIb**					
hsa-mir-449	5	54502117	54502207	-	
hsa-mir-449b	5	54502231	54502327	-	
hsa-mir-202	10	134911006	134911115	-	
hsa-mir-34b	11	110888873	110888956	+	
hsa-mir-34c	11	110889374	110889450	+	
hsa-mir-506	X	146119930	146120053	-	
hsa-mir-508	X	146126123	146126237	-	
hsa-mir-509	X	146149742	146149835	-	0.00001
hsa-mir-514-1	X	146168457	146168554	-	
hsa-mir-514-2	X	146171153	146171240	-	
hsa-mir-514-3	X	146173851	146173938	-	

*Whether each expression group was enriched in miRNAs located in genomic clusters was evaluated by Chi test. miRNAs in different subgroups were analyzed together in one test as long as they are located within a genomic cluster.

### Intronic miRNAs and their host genes have correlated expression patterns in normal tissues

It is believed that miRNAs positioned in introns frequently have the same expression patterns to their host genes, and data from meta-analysis and RT-PCR have been used to validate some of the genes [7,8]. We reasoned that our miRNA expression profiling could reproduce these observations.

We compiled a list of intronic miRNAs from a published literature based on the following rules modified from a previous report [7]: (1) the host gene and the miRNAs are transcribed from the same strand of DNA; (2) the host gene is a protein-coding gene with defined gene name and protein domains that link to its possible biological functions; (3) the miRNA does not have extra copies in other part of the genome since the transcription of each copy of the miRNA gene could be regulated by different mechanisms that would confound the result of our analyses. Among the 31 miRNAs qualified (Table 2), 22 of them had significant correlation (p < 0.05) with their host genes in expression among 19 tissue types. If the two miRNAs that had marginal correlation (p values between 0.05 and 0.07) was included (they could still be significant due to the use of different databases in this comparative study), total 77% of the miRNAs in our list had coherent expression patterns with their host genes. This result further corroborates the hypothesis that expression of intronic miRNAs is co-regulated with their host genes, and it also identifies the host genes that could be surrogate markers for expression of their intronic miRNAs.

**Table 2:** Correlation of expression patterns in human normal tissues between intronic miRNAs and their host genes.

**miRNA**	**Host Gene**	p value*	**Gene Description**
miR-106b	MCM7	0.047**	MCM7 minichromosome maintenance deficient 7 (S. cerevisiae)
miR-107	PANK1	0.022	pantothenate kinase 1
miR-126	EGFL7	0.027	EGF-like-domain, multiple 7
miR-128a	R3HDM1	0.002	R3H domain (binds single-stranded nucleic acids) containing 1
miR-128b	ARPP-21	0.001	cyclic AMP-regulated phosphoprotein, 21 kD
miR-139	PDE2A	0.007	phosphodiesterase 2A, cGMP-stimulated
miR-140	AIP2	**0.249**	WW domain containing E3 ubiquitin protein ligase 2
miR-148b	COPZ1	**0.472**	coatomer protein complex, subunit zeta 1
miR-149	GPC1	0.053	glypican 1
miR-151	PTK2	0.031	protein tyrosine kinase 2; focal adhesion kinase 1
miR-15b	SMC4L1	0.003	SMC4 structural maintenance of chromosomes 4-like 1 (yeast)
miR-186	ZNF265	**0.335**	zinc finger protein 265
miR-188	CLCN5	0.07	chloride channel 5 (nephrolithiasis 2, X-linked, Dent disease)
miR-190	TLN2	0.001	talin 2
miR-196b	HOXA9	0.005	homeo box A9
miR-204	TRPM3	0.009	transient receptor potential cation channel, subfamily M, member 3
miR-208	MYH6	6 × 10^-30^	myosin, heavy polypeptide 6, cardiac muscle, alpha
miR-211	TRPM1	0.004	transient receptor potential cation channel, subfamily M, member 1
miR-224	GABRE	0.006	gamma-aminobutyric acid (GABA) A receptor, epsilon
miR-25	MCM7	0.022	MCM7 minichromosome maintenance deficient 7 (S. cerevisiae)
miR-28	LPP	0.023	LIM domain containing preferred translocation partner in lipoma
miR-30e	NFYC	**0.618**	nuclear transcription factor Y, gamma
miR-326	ARRB1	0.013	arrestin, beta 1
miR-33	SREBF2	**0.287**	sterol regulatory element binding transcription factor 2
miR-335	MEST	0.006	mesoderm specific transcript homolog (mouse)
miR-338	AATK	0.0003	apoptosis-associated tyrosine kinase
miR-340	RNF130	**0.346**	ring finger protein 130
miR-342	EVL	0.017	Enah/Vasp-like
miR-346	GRID1	**0.895**	glutamate receptor, ionotropic, delta 1
miR-452	GABRE	0.0001	gamma-aminobutyric acid (GABA) A receptor, epsilon
miR-93	MCM7	0.022	MCM7 minichromosome maintenance deficient 7 (S. cerevisiae)

*When multiple clones are available in the database, the clone with the best p value was chosen. **Pearson correlation; bold-type numbers indicate those with p values > 0.07

### Combination of predicting transcription factor binding sites, sequence comparison, and expression analyses identifies candidate factors contributing to the tissue-specific expression of miRNA

**Figure 6:**
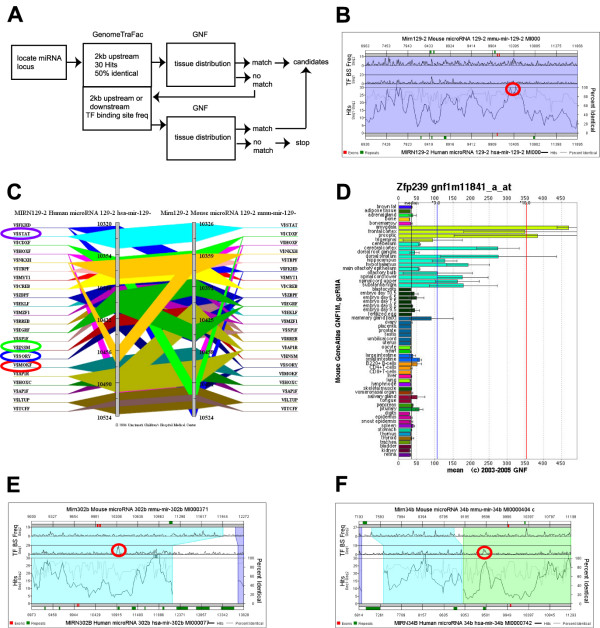
A flow chart outlines the search strategy used to identify candidate transcription factors that might be associated with the tissue-specific expression of miRNAs in the most differentially expressed groups (A). (B) A screen shot of the "regulogram" map from GenomeTraFac showed a peak for the "Hits" (red circle) upstream to the hsa-miR-129-2 (miR-129b) locus. (C) A screen shot of the transcription factor binding mapfrom GenomeTraFac showed four transcription factors with matching tissue distribution with that of hsa-miR-129b. STAT, STAT5; INSM, INSM1; SORY, SOX5; MOKF, MOK-2 (ZFP239). (D) A screen shot from the GNF database showed the expression of MOK-2 (ZFP239) in normal mouse tissues (no data availablefor human tissues). Two screen shots of the "regulogram" map from GenomeTraFac showed two regions of genomic sequences (red circles) close tothe hsa-miR-302b (E) and hsa-miR-34b (F) loci from which the binding sites for Nkx2-5 and FOXF2, respectively, were identified.

We have shown that most of the miRNAs in the same differentially expressed group are located within the same genomic clusters, suggesting the presence of common regulatory mechanisms to their expression. Genomic sequences flanking these clusters may contain regulatory elements that control expression of these miRNAs. Predicting candidate transcription factors that might be associated with tissue-specific expression of miRNAs would offer valuable information to elucidate how miRNAs participate in cell differentiation and tissue specification. Although it is difficult to distinguish whether the presence of a *cis*-regulatory element is truly functional or a stochastic event without performing experimental validation such as chromatin immunoprecipitation [20], it has been shown that evolutionarily conserved non-coding genomic sequences is more likely to have a functional role and a better source to search transcription factor-binding sites [22]. We sought to provide the proof of concept that a comparative genomics-based resource using human and mouse sequences such as GenomeTraFac [23] can detect putative *cis*-regulatory regions that may contribute to tissue-specific expression of miRNAs in some of the eight differentially expressed groups (Figure 6A).

We first tested the feasibility by examining the 2 kb-upstream sequence of the miR-1 cluster at chromosome 18 in the muscle-specific expression group because a number of myogenic factors are known to bind to the upstream sequences of muscle-specific miRNAs [20]. We found one peak larger than 30 "Hits" (defined by the number of transcription factor binding sites shared by human and mouse within a 200 bp window), the maximal "Hits" value from the software's graphic output, without particular surge of frequency of transcription factor-binding site in both human and mouse sequences (see the left "Hits" peak circled with red in the upper part of the Additional file 5). The transcription factor-binding site prediction showed two MyoD-binding sites in this region. Another region close to the edge of this 2 kb segment had another larger-than-30 "Hits" peak but no MyoD-binding site was seen. There were another two peaks with "Hits" close to 30 about 500 bp downstream to the MyoD-binding site-containing peak that actually had two MyoD-binding sites (one for each, data not shown). We reasoned that prioritizing the genomic sequences for subsequent analysis would be critical if the transcription factor of our interest is unknown (unlike MyoD in this test case), so we chose the sequence with "Hits" larger than 30 as the first criteria for transcription factor-binding site prediction. The sequences from the graphic output generally showed larger than 40% identical between human and mouse, so we set 50% identical between human and mouse as a threshold. Expression of the transcription factors binding to the predicted sites was further examined in a host of more than 60 normal human tissues from the database of the Genomics Institute of the Novartis Research Foundation (GNF) [24], and the transcription factors with the same tissue distribution as the tissue-specific miRNAs would conceivably be a favorable (certainly not only) target for future validation. We applied this searching strategy to the three miRNAs in the group III (brain) and identified a zinc finger protein, MOK-2 (ZFP239) (Figure 6B to 6D). Our heat map in Figure 5 also showed low but detectable miR-129 in PBMC, testes, and pancreas, and interestingly in this segment of sequence we identified binding sites for STAT5, SOX5, and INSM1 that are specifically expressed in these three tissues, respectively (see Additional file 6).

This searching strategy would likely fail if the transcription factor-binding sites are not conserved between human and mouse although sequence homology in general is acceptable (for example, 50% identical). This was best represented by the search of transcription factors for the group V and group VIII miRNAs. In the case of group V, the upstream sequence of the miR-302b locus had a peak of "Hits" but no binding site for heart-specific factors could be found from that region. However, a region closer to miR-302b showed a significant spike in binding-site frequency despite the very low "Hits" (Figure 6E). Note that the "Hits" and the transcription factor-binding site frequency for mouse sequence in this region were extremely low. Examining this DNA segment indeed showed four binding sites for the heart-specific transcription factor Nkx2-5 (see Additional file 7). GenomeTrafac lacks data for most of the miRNAs in groups VII and VIII, so we examined the miR-34b cluster that had prominent expression in testes as well as in lung. In the region upstream to the miR-34b locus, a segment with a spike of transcription factor-binding site frequency in human sequence (in this case a small spike in mouse sequence too) and 25 "Hits" (Figure 6F) was examined and a forkhead transcription factor, FOXF2, had a matching expression pattern with miR-34b in lung and placenta (see Additional file 7). The binding site for SOX5 (a testis-enriched factor, see Additional file 7) was present at the binding-site peak region downstream to the miR-34b locus, suggesting that both upstream and downstream sequences to the transcript-start site should be examined.

Our results suggests that, using the GenomeTraFac web tool, we can identify several candidate transcription factors that may participate in tissue-specific expression of miRNAs by the following workflow (Figure 6A): (1) Start from the 2 kb sequence upstream to the start of the miRNA transcript, and start from the regions with peaks of "Hits" larger than 30. (2) Examine the tissue distribution of the transcription factors that bind to the regions in comparison to the miRNA expression patterns. (3) If there is no matched tissue distribution, repeat the search in the 2 kb sequence downstream to the start of the transcript. (4) If the transcription factor-binding sites are not conserved between human and mouse, look for regions with low "Hits" but with increased transcription factor-binding site frequency in human, followed by examining the tissue distribution.

### Predicted target genes of miRNAs with reduced expression in brain and peripheral blood mononuclear cells identifies a list of genes essential for development of these two tissue types in mouse models

Identification of genes whose expression is regulated by miRNAs provides a lead for the functional roles of miRNAs, and predicting target genes of the tissue-specific miRNAs identified in our differentially expressed groups would greatly facilitate understanding the miRNA-regulated biological correlates of those tissues. One example is the group IVb miRNAs that had almost no expression in brain/PBMC compared to the rest of tissues that invariably had moderate to high abundance (Figure 5). Since current evidence supports a general notion of opposite expression levels between a miRNA and its target genes in tissues [25], it is possible from this pattern of tissue distribution that we may identify a list of genes targeted by these miRNAs with suppressed expression in all tissues but brain and PBMC.

One member of the miR-199a (miR-199a-2) is located at only 5.6 kb away from miR-214, while miR-199a-1 and miR-199b are located at two separate regions with no other miRNAs nearby; miR-10a and miR-143 do not have relationship with miR-199a/199b/214 in genomic structure and were excluded from the analysis. To validate the low abundance of miR-199a/199b/214 in brain, their expression was examined in 6 additional adult brain specimens (including four derived from different regions of the brain and one fetal brain specimens) and all were reproducibly lower than the other tissue types (Table 3). Lower expression of miR-199a/214 was previously reported in brain than in liver, thymus, testes, and placenta by 16 to 180 folds in a study using microarrays [16]. Interestingly, expression of miR-199a/214 in brain compared to other major tissues was also reduced during zebrafish embryonic development [26].

**Table 3:** The abundance of miR-199a/199b/214 in fetal/adult brain and non-brain/PBMC specimens (by average C_T_)

Tissues/C_T_/miRNAs	hsa-miR-199a	hsa-miR-199b	hsa-miR-214
Ave non-brain/PBMC*	27.9	28.2	26.9
Fetal Brain	31.2	33.6	30.7
Brain 1	33.3	33.8	32.2
Brain 2	32.8	33.3	31.8
Brain 3	33.1	34.0	32.2
Frontal Cortex	33.6	35.0	32.2
Cerebellum	32.3	33.2	31.3
Occipital Cortex	34.2	35.0	32.9
Striatum	33.9	33.6	32.7

*p values between non-brain/PBMC tissues and all brain specimens by Student's *t *test: miR-199a, 4.1×10-8; miR-199b, 3.6×10-12; miR-214, 1.5×10-10

**Figure 7:**
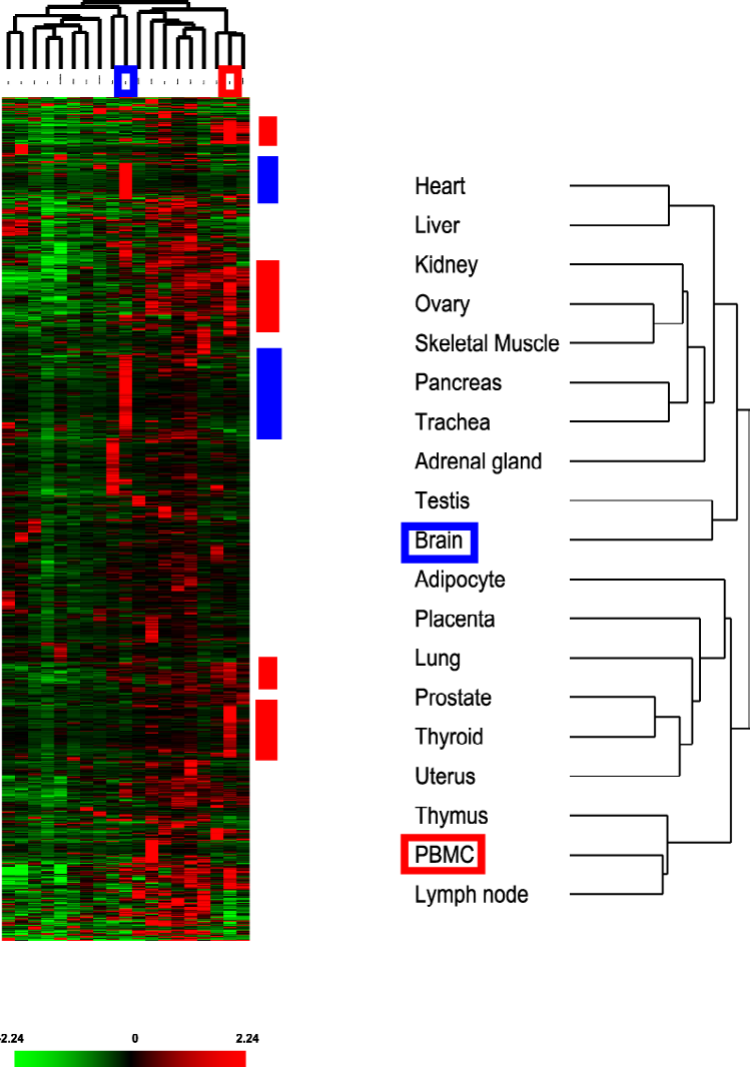
The list of predicted target genes for miR-199a/199b/214 was refined by their expression in 19 normal tissue types extracted fromthe GNF database. Blue bars on the right side of the heat map, genes with brain-specific expression; red bars, genes with PBMC-specific expression. The pseudocolor scale represents the gene expression level that has been transformed to the log2-based ratio to the average signal of all genes extracted.

All target genes (N = 1939) for miR-199a/199b/214 predicted by the miRanda web tool from miRBase [27] were combined, and their expression in 19 tissue types extracted from the GNF database (see Methods) was examined. To focus on more differentially expressed genes, 1125 genes with variation in expression among tissues equal or larger than 3.24 fold (1.8 under log_2 _base) were selected for analysis. Unexpectedly, almost all genes with reduced expression in non-brain and non-PBMC tissues did not show simultaneous increased expression in brain and PBMC; they rather showed elevated expression in either one or the other (Figure 7). To reduce the chance of selecting genes with stochastic increased expression in brain or PBMC, we selected genes that only agglomerated in unique expression clusters. There were 168 genes with appreciable overexpression in brain, and 146 genes were overexpressed in PBMC, whereas there were only 2 genes had high expression in both tissues. Based upon these expression patterns, these 314 genes (28% of genes initially selected for analysis) formed a more refined list of candidates than the originally predicted target genes by miRanda. Many of these 314 genes are required by the nervous and hematopoietic systems in development as well as in adult. The opposite expression pattern between miR-199a/199b/214 and their 168 refined predicted targets in fetal/adult brain and non-brain tissues strongly suggested that repressed expression of these three miRNAs is important in brain development. One way to ultimately test his hypothesis is to introduce loss-of-function mutations of these genes in a mouse embryo.

International Gene Trap Consortium (IGTC) maintains a database to curate functions of genes across the mouse genome by gene trapping that is a high-throughput approach to introduce insertional mutations and generate loss-of-function alleles in embryonic stem cells [28]. There were 31 out of the 168 predicted brain targets that had available phenotypic information in this database, and 23 of the 31 genes had either defect in the nervous system or in behavioral/neurological functions (Table 4 and see Additional file 8). To assure that such genes are not overrepresented in the IGTC database, 33 genes that had phenotypic information available were randomly selected from 6991 genes recorded in the database as of Feburary 13^th^, 2007, and only 7 genes had neurological defects when mutated, indicating that functions in the nervous system is indeed the most enriched category among these 31 genes (p = 2 × 10^-12 ^by Chi test). The same strategy was used to evaluate the functional categories of the 146 predicted PBMC targets that had 30 genes with available phenotypic records in the database. Instead of 9 of the 33 randomly selected genes with defects in the immune and/or hematopoietic systems, 14 of the predicted PBMC genes had phenotypic changes in these two systems, which confirmed a moderate but still significant enrichment of genes in this functional category (p = 0.002).

**Table 4:** Phenotypic changes in mice carrying loss-of-function mutations of the predicted brain and PBMC target genes.

Phenotype	Brain	PBMC	Random
Nervous system^1^	21	5	5
Immune system^2^	2	8	7
Nervous/immune^3^	2	6	2
Embryonic lethal^4^	2	0	4
Other systems^5^	3	4	11
Normal^6^	1	7	4

Total gene number	31	30	33

1 Phenotype in nervous system, including behavioral and neurological defects, but no defects in immune and hematopoietic system.

2 Phenotype in immune system, including the hematopoietic system, but no nervous system or behavioral/neurological defects.

3 Phenotype from both 1 and 2 can be seen.

4 Embryonic lethal with no other phenotypic records, indicating that the embryo dies too early to observe meaningful phenotype.

5 Defects in other tissues/organs/systems than nervous and immune systems.

6 The phenotypic record showed no defect was seen.

Our refined 314 predicted targets could be more enriched than we observed in the functional categories of nervous and immune systems because the IGTC database lacks phenotypic records for most of these genes, and roles of many of them (for example, interleukins, tumor necrosis factors, ion channels, and neurotransmitter transporters) in these two systems have been well documented.

## Discussion

### Our miRNA expression profiles provide comprehensive information about general abundance as well as tissue-specificity of miRNAs

In this study, we examined the expression of miRNAs in a comprehensive list of normal human tissues using 345 unique miRNA assays, and identified miRNAs that were expressed in specific tissues with minimal or no expression in other tissues we examined, such as miR-129/219/330 in brain, miR-124a/124b in brain and PBMC, and two groups of miRNAs primarily expressed in placenta and testes. We were also able to identify miRNAs with moderate to high expression in all tissues examined except for certain organs that had much lower or no expression at all, such as miR-199a/199b/214 in brain and PBMC and miR-10a/10b in brain.

Our study provided an opportunity to re-visit and confirm the tissue-specific miRNAs previously reported in literature. For example, liver-specific miR-122a had significant expression in liver [13] and regulated cholesterol in plasma [19]; we indeed saw no expression in all non-liver tissues except for brain (34 copies) and thymus (19 copies) that had much less expression than liver (185-fold and 332-fold less, respectively). One of the brain-specific miRNAs, miR-219, described in a previous report [13] was also confirmed with our data, in which brain had 85 copies and merely 5 copies were found in PBMC, and no expression detected in the rest of the tissues.

For reasons such as limited types of tissues and number of miRNAs examined and sensitivity of the assays, several miRNAs that were previously considered as "tissue-specific" actually showed significant expression in other tissues from our data, and therefore their tissue distribution needs to be redefined. For example, one "brain-specific" miRNA, miR-124 [29], was described in a number of reports and was demonstrated to shift the expression profiles of HeLa cells towards the brain signature [30,31]. Although our data did show most abundant expression of this miRNA in brain and no expression in most of the tissues surveyed, it also had significant expression in PBMC (from 1/4 to half of what was detected in brain) and detectable in thymus and one of the testes (from 50- to 100-fold less than what was in brain). It would be more reasonable to define miR-124a/b as preferentially expressed in brain and PBMC.

In some cases, a broader tissue distribution for miRNAs described from our data suggested that they might have more functions than what was originally described. For example, miR-375 that was identified as "pancreas islet-specific" miRNA and functions as a regulator of insulin secretion from the islet cells [32], but we clearly showed that this miRNA belong to the epithelial subgroup of the GI/epithelial expression cluster, and was preferentially expressed in organs lined with epithelium (Group III in Figure 3). It was proposed by the authors that miR-375 could be a pharmacological target for treating diabetes [32], but the possibility of its functions in non-insulin-secreting epithelial cells suggested by our data should not be dismissed, especially any possible collateral effects from other tissues when targeting miR-375 for pancreas treatment is considered.

Not having enough tissue types for expression profiling might cause incorrect denotation of tissue-specific expression. For example, miR-134 was found to regulate development of dendritic spines of neurons, and regarded as "brain-specific" [33], whereas our data clearly demonstrated its expression in many other tissues with similar (placenta and testis) or even higher (adrenal gland) expression levels. We also discovered that another two "brain-specific" miRNAs, miR-135 and miR-183, identified previously [13] were more abundant in several non-brain tissues that were not examined in their original report.

### Universally expressed miRNAs are candidates participating fundamental metabolic pathways in normal cells

We identified a group of 15 miRNAs (see Additional file 1) that are universally expressed at similar levels in normal tissues we examined based upon their C_T _values and the variability of their C_T _values among samples, and the scope of our tissue collection suggests that these miRNAs might exhibit similar expression patterns in tissues we did not examined. Such a feature characterizes these miRNAs as a candidate of universal reference to normalize miRNA expression in normal human tissues, as we did in our analysis using 4 miRNAs from this list.

The expression pattern and tissue distribution of these 15 miRNAs suggests that they might be associated with fundamental functions, such as metabolism, required for normal human cells. For example, miR-29b was found to control the amount of the branched-chain α-ketoacid dehydrogenase complex that catalyzes the first irreversible step in branched-chain amino acid catabolism [34]. Furthermore, deregulated expression of these universally expressed miRNAs could also link to pathological states of a cell, such as neoplastic processes. Differential expression of some of them in tumors has been demonstrated, for example, miR-15/16 in chronic lymphocytic leukemia [11], miR-92 in lymphoma and lung cancer [35], and miR-140 in pancreatic cancer [36].

### The clustering patterns of normal tissues by miRNA and mRNA expression profiles are similar

Even with much less degree of freedom than mRNAs, miRNA expression profiles reflect the developmental lineage and differentiation state of cells and successfully classified poorly differentiated tumors that could not have definitive diagnosis by histopathology, while the classification based upon the mRNA profiles was highly inaccurate [14]. For this reason, one might anticipate that miRNA expression profiles would classify normal human tissues better than the mRNA profiles as well. To our surprise, the clustering of tissues using the miRNA expression profiles was very similar to that obtained by the mRNA expression profiles. One possible explanation is that miRNAs preserve more of the "cellular identity signature" compared to mRNAs under the genomic instability and heterogeneity that characterize neoplastic cells, whereas in normal tissues such a variable environment does not exist so the performance of both miRNA and mRNA expression profiles on tissue classification is comparable.

The uniqueness of the tissue clustering using the miRNA expression profiles compared to that with the mRNA profiles appeared to be contributed by several groups of miRNAs with distinctive expression patterns, such as the ones in epithelial tissues, and those in placenta, testes, brain, and PBMC. The distinctive expression patterns in turn highlight the central roles played by miRNAs in histogenesis of epithelial tissues and in key physiologies of placenta, testis, brain, and the hematopoietic system. The group of epithelial miRNAs is probably the best example to support the concept of "cellular identity signature" since they also have marked expression in cancer cell lines derived from epithelial tissues [37].

Because a considerable portion of miRNAs had tissue-specific expression patterns and the average miRNA copy numbers in all tissues were highly variable, global normalization that assumes total abundance of the transcripts from all genes is constant across different tissues and is frequently used for normalization of mRNA expression data does not appear to be appropriate to normalize miRNA expression data. This characteristic of miRNA expression patterns among tissues we observed underscores the earlier findings in which total abundance of miRNAs was altered in tumors [14] as well as in Dicer-knockout animal models [38,39].

### MicroRNA genes localized within a genomic cluster are preferentially co-expressed as a "transcription unit"

Chromosomal abnormalities such as deletion/amplification of genes or loss/gain of chromosomes are characteristics of neoplastic cells, and such features could influence expression of genes within such afflicted regions that at least some of these genes show a coherent expression pattern, and this can be identified as distinctive expression clusters when global profiles of mRNA expression are analyzed by hierarchical clustering. For example, epidermal growth factor receptor (EGFR) is amplified in about 40% of GBM, and gene expression profiling of GBM revealed that EGFR and its neighboring genes were tightly clustered together and had substantially increased expression in the tumors that had EGFR amplification [40]. In the same study, a cluster of six C-C motif-containing cytokines located at Chromosome 17q12 within a 100 kb region (with spacing from 3.5 kb to 35 kb between them) could also be identified with a coordinated expression pattern, suggesting the presence of a co-regulated mechanism of transcription of these genes [40]. However, in most cases mRNAs in a hierarchical cluster with highly correlated expression are not mapped to the same genomic regions. This provides a striking contrast to what was observed in our miRNA expression profiles, in which most miRNAs with similar expression patterns are encoded from the same genomic region (usually with spacing from 1 kb to 5 kb between them). One possible explanation for this is that most miRNA genes located within the same genomic cluster are encoded in a polycistronic structure so they are synthesized, processed, and mature to final products in a parallel fashion. Conversely, miRNAs within the same genomic region that share common expression pattern would predict that these miRNA genes are transcribed as a polycistron.

There were some exceptions in which miRNAs that are localized in the same genomic cluster but did not show coherent expression patterns in our data. This might be caused by different regulatory mechanisms for the transcription of these miRNAs, or these miRNAs share the same transcript (polycistronic) but differential control of the maturation process for each miRNA in the same transcript determines their final abundance.

### Target genes predicted from miRNAs with low expression in brain and peripheral blood mononuclear cells are candidates required for development and maintenance of these two tissue types

It is believed that miRNAs down regulate the steady-state levels of their target mRNAs, which has been demonstrated in cell lines or entire organisms by examining limited number of miRNAs [19,30,41,42]. The major drawback of these studies is that they investigated the interaction between miRNAs and their target genes by either overexpression or knockdown experiments, since such an unnatural expression levels of miRNAs might cause artifacts. The only study that investigates the association of expression between miRNAs and their targets in normal and neoplastic specimens derived from a spectrum of tissue types without modifying their expression was using "tissue-specific" miRNAs extracted from other sources and analyzed the expression of predicted target genes of these miRNAs using published microarray datasets [25]. We used a comprehensive list of tissue specimens and highly quantitative miRNA assays to identify several groups of miRNAs that had specific expression in certain tissues, and examined the expression of their predicted target genes in the same tissue types from public microarray database.

We initially expected to observe complementary expression patterns of miRNAs and their predicted target genes among the tissues, but in the case of miR-199a/199b/214 that had low expression in brain and PBMC compared to the rest of tissues, most predicted targets that showed brain/PBMC-specific expression only appeared in either brain or PBMC but hardly both. Similarly, expression of most predicted genes of miR-129/219/330 (higher expression in brain) had decreased expression in brain, but their expression in non-brain tissues was highly variable. It appears that expression of miRNA has a binary effect on expression of its targets, in that the suppression of its target by the miRNA is predominant when miRNA expression is high, whereas when the miRNA expression is reduced, other tissue-specific parameters such as transcription factors serve as a different level of gene expression control. This is supported by the observation of the same binary patterns in the expression of predicted miRNA target genes in our data from other tumor specimens and cell lines [37].

High-throughput identification of miRNA target genes could potentially rely on either manipulating expression of miRNAs (overexpression or knock-down) and examining resulting changes of gene expression profiles in cells/tissues using microarrays, or algorithm prediction followed by *in vitro *validation. Although *in silico *prediction appears to circumvent the time and cost issues associated with the transfection/knock-down experiments and microarrays, a long list of predicted output could be frustrating for investigators to focus on a few candidates for validation. Furthermore, genes predicted as targets might not be biologically meaningful if the miRNAs and their predicted target genes are never expressed in the same tissues. In our study, we filtered the original list of predicted targets by comparison of the tissue distribution between miRNAs and their target genes. This strategy seemed to produce a much-focused group of genes corresponding to the physiological functions of the organs. For example, many predicted target genes of miR-199a/199b/214 (low expression in brain and PBMC) are required by the developing nervous and hematopoietic systems.

## Conclusion

Our data and analyses of expression patterns presented a global view of tissue distribution of miRNAs and the relation to their chromosomal locations. We presented evidence that such data support identification of specific miRNAs as markers to correlate with the functions of normal or disease tissues in which these miRNAs are expressed, and identification of the predicted miRNA target genes that are required in the developing nervous and hematopoietic systems. We also demonstrated a proof-of-principle strategy using the GenomeTraFac web source to precede future experimental validation for identifying candidate transcription factors associated with tissue-specific expression of miRNAs.

## Methods

### Total RNA samples

Total RNA samples of normal human tissues from commercial sources were purchased from Ambion (Austin, TX), Stratagene (La Jolla, CA), and BD Biosciences (Mountain View, CA).

### Quantitation of miRNAs

TaqMan^® ^MicroRNA Assays were used to quantitate miRNAs in all of our studies according to the conditions published previously [17]. In brief, each 7.5 μl RT reaction contained purified 3.75 ng of total RNA, 50 nM stem-loop RT primer (Applied Biosystems, Foster City, CA), 1×RT buffer (Applied Biosystems), 0.25 mM each of dNTPs, 3.33 U/μl MultiScribe™ reverse transcriptase (Applied Biosystems) and 0.25 U/μl RNase inhibitor (Applied Biosystems). The reactions were incubated in an Applied Biosystems 9700 Thermocycler in a 384-well plate for 30 min at 16°C, 30 min at 42°C, followed by 5 min at 85°C, and then held at 4°C. RT products were diluted three times with H_2_O prior to setting up PCR reaction. Each real-time PCR for each microRNA assay (10 μl volume) was carried out in quadruplicate, and each 10 μl reaction mixture included 2 μl of diluted RT product, 5 μl of 2×TaqMan^® ^Universal PCR Master Mix, 0.2 μM TaqMan^® ^probe, 1.5 μM forward primer, and 0.7 μM reverse primer, respectively. The reaction was incubated in an Applied Biosystems 7900HT Fast Real-Time PCR System in 384-well plates at 95°C for 10 min, followed by 40 cycles of 95°C for 15 sec and 60°C for 1 min. The threshold cycle (C_T_) is defined as the fractional cycle number at which the fluorescence exceeds the fixed threshold of 0.2. Automated multi-well distribution of samples was done using the HYDRA^® ^II PLUS-ONE System (Matrix Technologies, Hudson, NH).

### Data adjustment and filtering for hierarchical clustering

Four human miRNAs (miR-30e, miR-92, miR-92N, and miR-423) that were least variable among the 40 normal tissues in this study were identified, and the average quantity of these four in each tissue was used to normalize the RNA input. Normalized data from assays with C_T _values greater than 35 were treated as 35 and were subject to hierarchical clustering by two ways. One is mean-centering data for each miRNA but not tissues, followed by correlation similarity metrics for both miRNA and tissue clustering (Figure 2); the other was to use Euclidean similarity metric and correlation similarity metric to cluster miRNAs and samples, respectively, without centering the data (Figure 3). We also normalized the sample input by quantitating small nuclear RNAs using the TaqMan^® ^MicroRNA Assay Controls (Applied Biosystems), and the key patterns of the hierarchical clustering of both miRNAs and tissues were very similar (data not shown, and see Additional file 1 for data normalized by small nuclear RNAs). The copy number of miRNAs in each cell (assuming each cell contains 30 pg of total RNA) was calculated from a formula 10(40−CT)/3.34/22
 MathType@MTEF@5@5@+=feaafiart1ev1aaatCvAUfKttLearuWrP9MDH5MBPbIqV92AaeXatLxBI9gBaebbnrfifHhDYfgasaacH8akY=wiFfYdH8Gipec8Eeeu0xXdbba9frFj0=OqFfea0dXdd9vqai=hGuQ8kuc9pgc9s8qqaq=dirpe0xb9q8qiLsFr0=vr0=vr0dc8meaabaqaciaacaGaaeqabaqabeGadaaakeaacqaIXaqmcqaIWaamdaahaaWcbeqaaiabcIcaOiabisda0iabicdaWiabgkHiTiabboeadnaaBaaameaacqqGubavaeqaaSGaeiykaKIaei4la8IaeG4mamJaeiOla4IaeG4mamJaeGinaqdaaOGaei4la8IaeGOmaiJaeGOmaidaaa@3D2A@ that was estimated using synthetic lin-4 miRNA [17].

When gene expression data were extracted from the GNF [43] database, the signal intensity of each gene was divided by the average of signal intensities of all genes extracted, followed by log_2 _transformation. Data adjustment for centering and similarity metric for hierarchical clustering was the same as described above. Differentially expressed genes were selected to avoid spurious clustering results by removing genes with variation in expression among tissues less than 1.8 (under log_2 _base). Higher cutoff will generate less number of genes and therefore was avoided. Genes from the Stanford GBM database were selected if they had analyzable data in more than 80% of the samples among the tissues examined.

### Extraction of data from public databases

Genomic sequence of miRNA cluster and the predicted transcription factor binding sites were extracted from the "*Cis*-element clusters within BlastZ Aligments" option at the GenomeTraFac [44]. Tissue distribution of selected genes was derived from the GNF database [43], in which the expression data from the same tissue types examined in our study were extracted for analyses. Target genes of miRNAs were predicted using the miRanda open-source software [45] associated with the miRBase [46] with a cutoff p value less than 0.05. The IGTC database [47] was used to search the phenotype of mice carrying loss-of-function mutations.

### Statistical analyses

The correlation coefficient (*r*) between repeating specimens and the correlation of expression between intronic miRNAs and their host genes (threshold p value was set to 0.05) were analyzed using Pearson regression. Comparison of mean was evaluated using 1-tail Student's *t *test with unequal variance. Chi test was used to analyze the significance of genomically clustered miRNAs in each differentially expressed group: the expected frequency was the number of all miRNAs among the 345 miRNAs examined that are located within the genomic clusters carrying the genomically clustered miRNAs regardless whether or not they were in the differentially expressed groups.

## Abbreviations

miRNA, microRNA; PBMC, peripheral blood mononuclear cells; GNF, Genomics Institute of the Novartis Research Foundation; IGTC, International Gene Trap Consortium.

## Authors' contributions

YL designed experiments, performed assays, analyzed data, and wrote the manuscript; DR and LW performed assays; CC designed assays and edited the manuscript.

## Supplementary Material

Additional file 1 Complete data of miRNA expression in 40 normal human tissues. Complete data normalized by the least variable miRNAs and small nuclear RNAs. Click here for file

Additional file 2 The clustering patterns of normal human tissues using mRNA expression profiles. The clustering patterns of normal human tissues using mRNA expression profiles taken from Shyamsunder et al. [18]. Click here for file

Additional file 3 Differential abundance of miRNAs in normal human tissues. A color-coded diagram illustrates the differential abundance of miRNAs in normal human tissues. Normal tissues were generally arranged by their positions in human body, as highlighted at the right side of the diagram, whereas miRNAs were sorted based upon their annotated ID. Click here for file

Additional file 4 Estimated copy numbers of miRNAs in normal human tissues. Complete data of estimated copy numbers of miRNAs in normal human tissues transformed from the Additional file 1. Click here for file

Additional file 5 The "regulogram" of the genomic sequence close to the hsa-miR-1-2 locus where MyoD binding site was identified. The "regulogram" from GenomeTraFac showed the genomic sequence close to the hsa-miR-1-2 locus where MyoD binding site was identified. Click here for file

Additional file 6 Expression patterns of INSM1, STAT5, and SOX5 in normal human tissues. Expression patterns of INSM1, STAT5, and SOX5 in normal human tissues from the GNF database. Click here for file

Additional file 7 Binding sites for Nkx2-5, SOX5, and FOXF2 and their tissue distribution. Binding sites for Nkx2-5, SOX5, and FOXF2 from GenomeTraFac, and their tissue distribution from the GNF database. Click here for file

Additional file 8 Phenotypic data for the refined list of predicted target genes of miR-199a/199b/214. Complete phenotypic data extracted from IGTC for the refined list of predicted target genes of miR-199a/199b/214. Click here for file
