# Supplementary figures and images for: Characterization of microRNA expression profiles in normal human tissues
Source: BMC Genomics. 2007 Jun 12;8:166. doi: 10.1186/1471-2164-8-166 (PMC1904203; doi:10.1186/1471-2164-8-166)

# Additional Data File 2

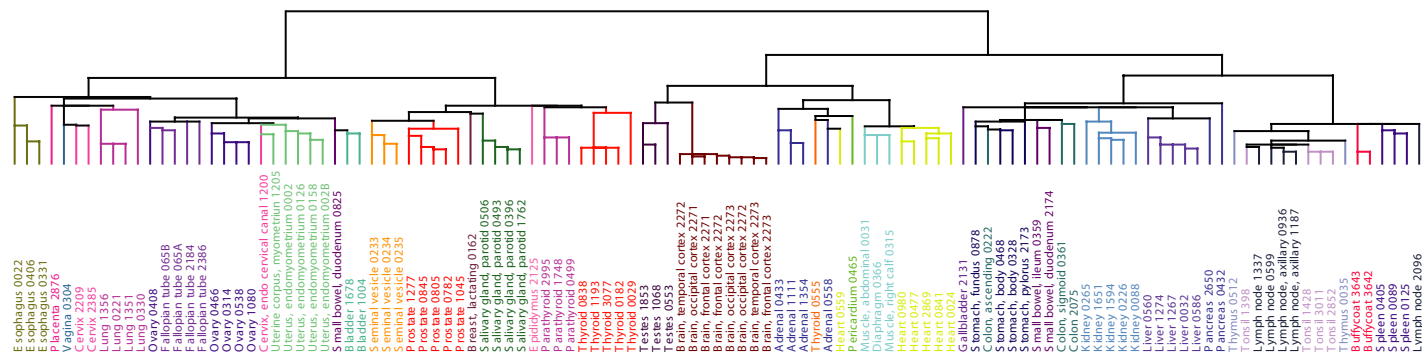

Supplement: Additional file 2 — The clustering patterns of normal human tissues using mRNA expression profiles. The clustering patterns of normal human tissues using mRNA expression profiles taken from Shyamsunder et al. [18]. [file 1471-2164-8-166-S2.pdf]

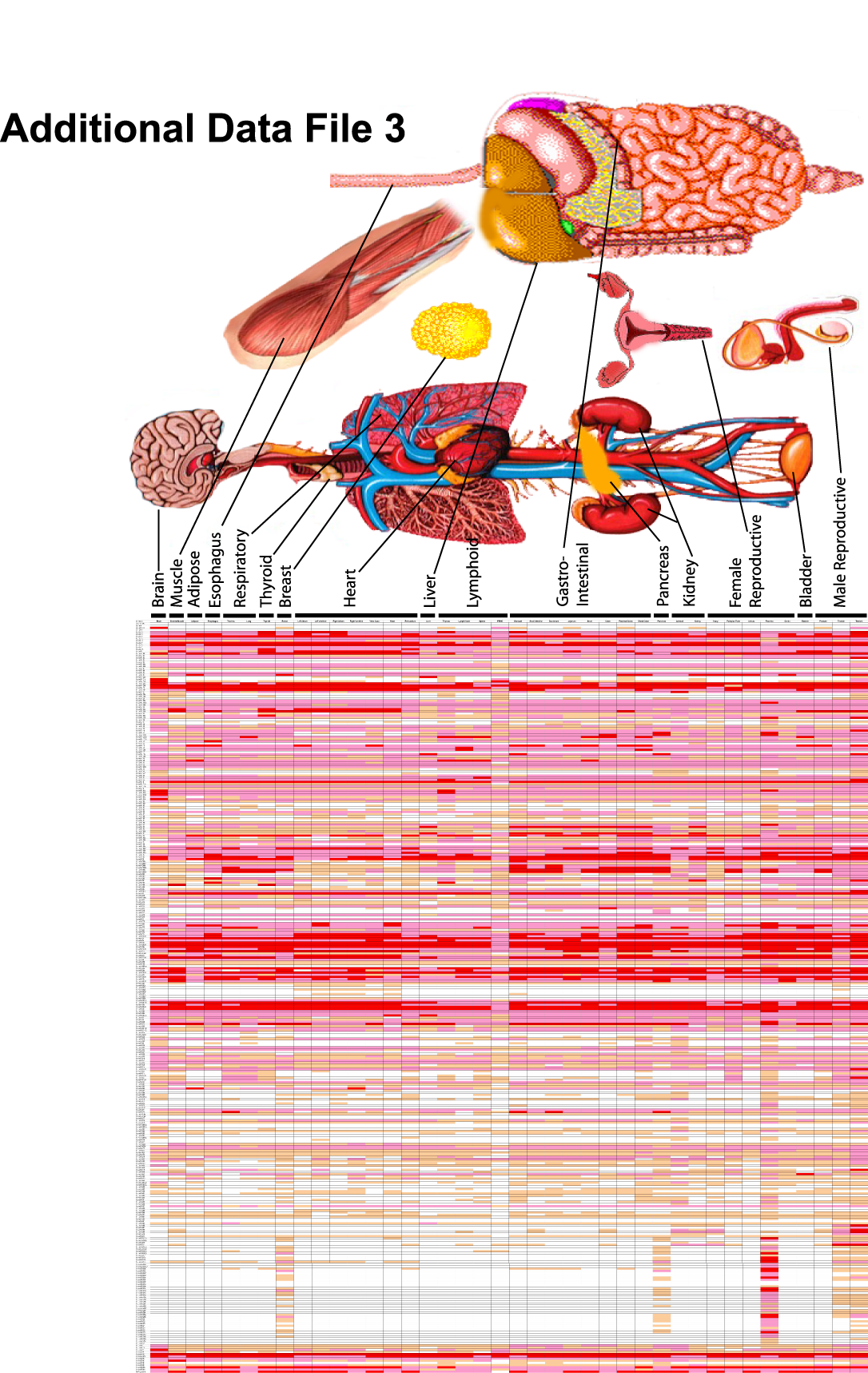

Supplement: Additional file 3 — Differential abundance of miRNAs in normal human tissues. A color-coded diagram illustrates the differential abundance of miRNAs in normal human tissues. Normal tissues were generally arranged by their positions in human body, as highlighted at the right side of the diagram, whereas miRNAs were sorted based upon their annotated ID. [file 1471-2164-8-166-S3.tiff]

# Additional Data File 6

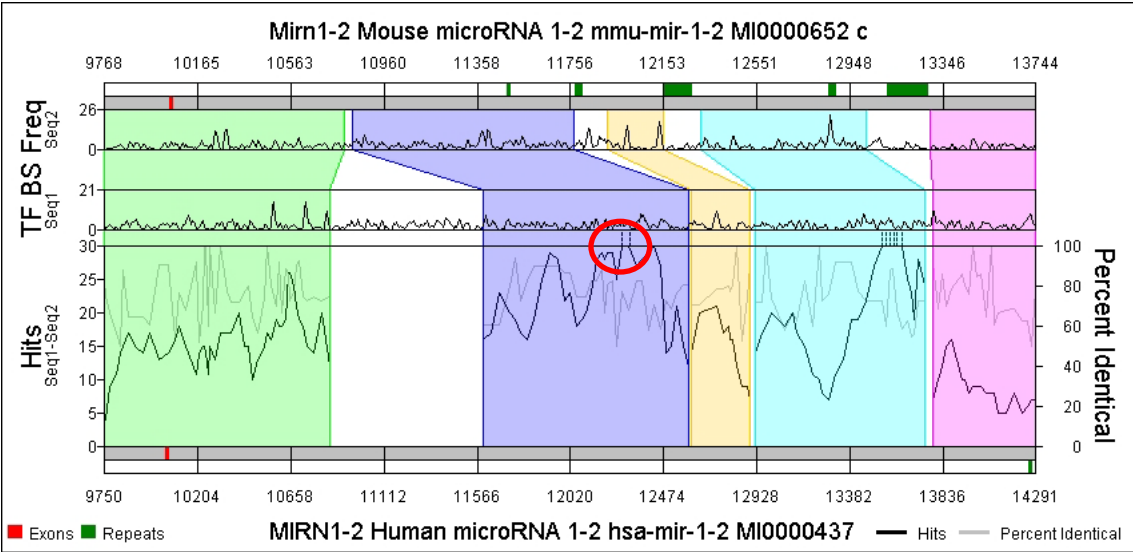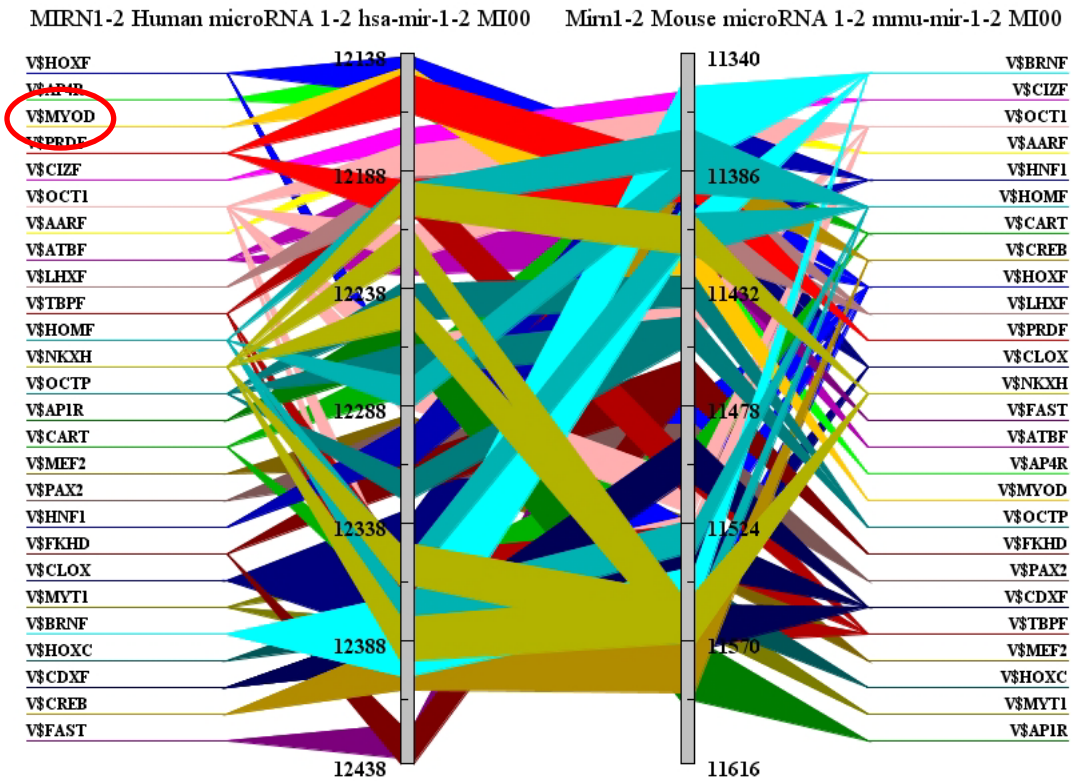

Supplement: Additional file 5 — The "regulogram" of the genomic sequence close to the hsa-miR-1-2 locus where MyoD binding site was identified. The "regulogram" from GenomeTraFac showed the genomic sequence close to the hsa-miR-1-2 locus where MyoD binding site was identified. [file 1471-2164-8-166-S5.pdf]

# Additional Data File 7

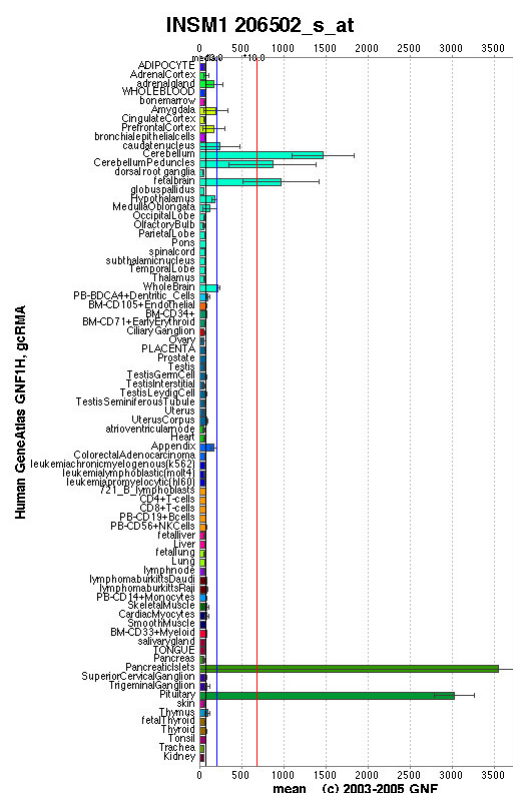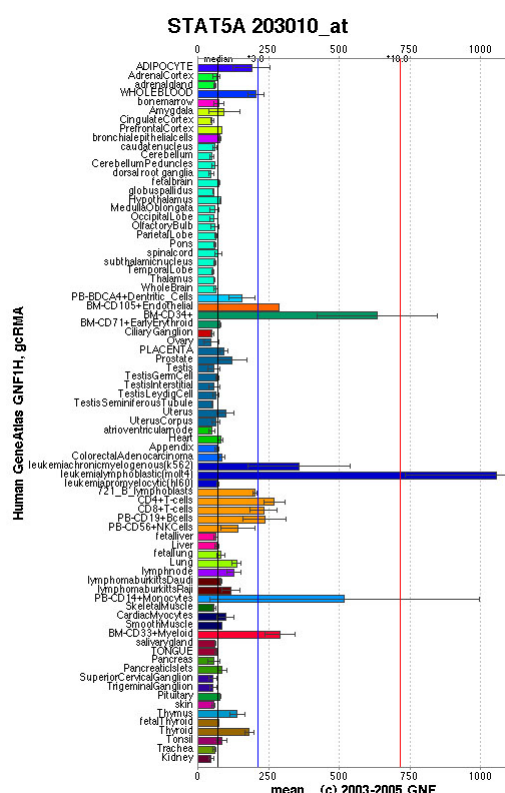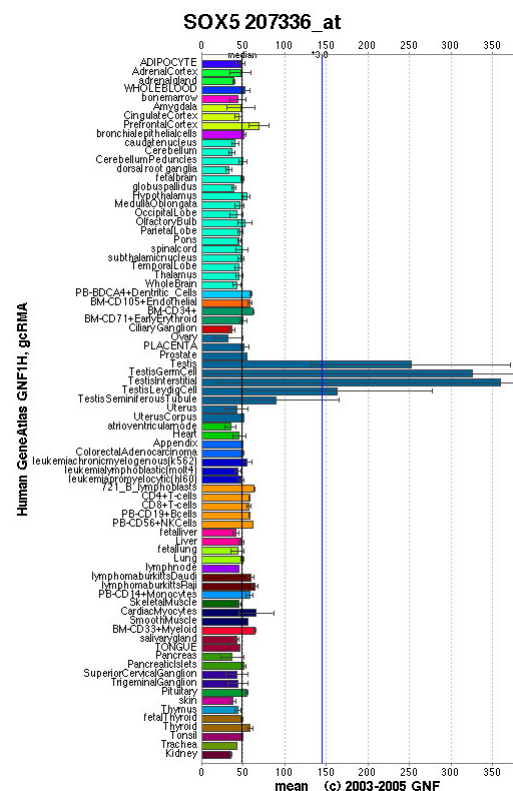

Supplement: Additional file 6 — Expression patterns of INSM1, STAT5, and SOX5 in normal human tissues. Expression patterns of INSM1, STAT5, and SOX5 in normal human tissues from the GNF database. [file 1471-2164-8-166-S6.pdf]
