# Supplementary material for: Characterization of microRNA expression profiles in normal human tissues
Source: BMC Genomics. 2007 Jun 12;8:166. doi: 10.1186/1471-2164-8-166 (PMC1904203; doi:10.1186/1471-2164-8-166)

Additional Data File 8

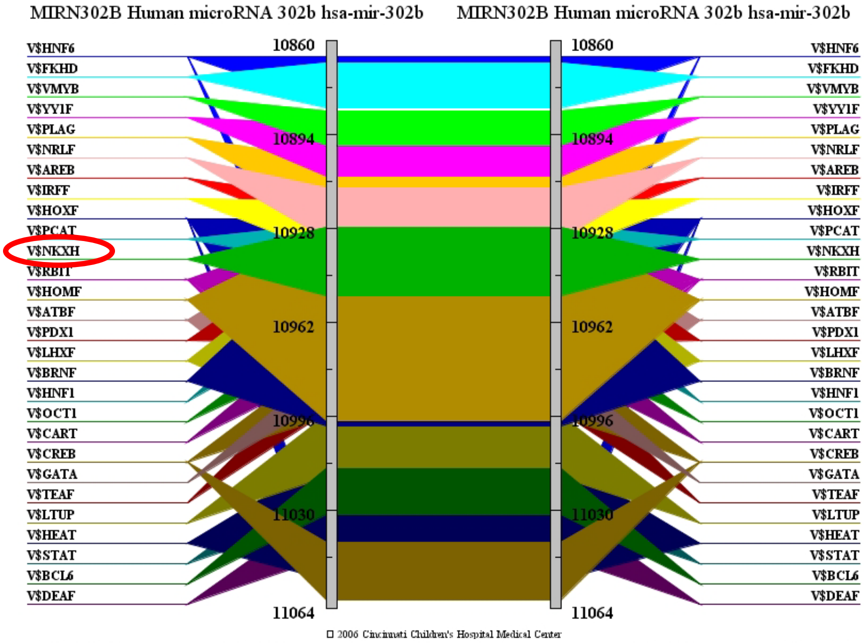

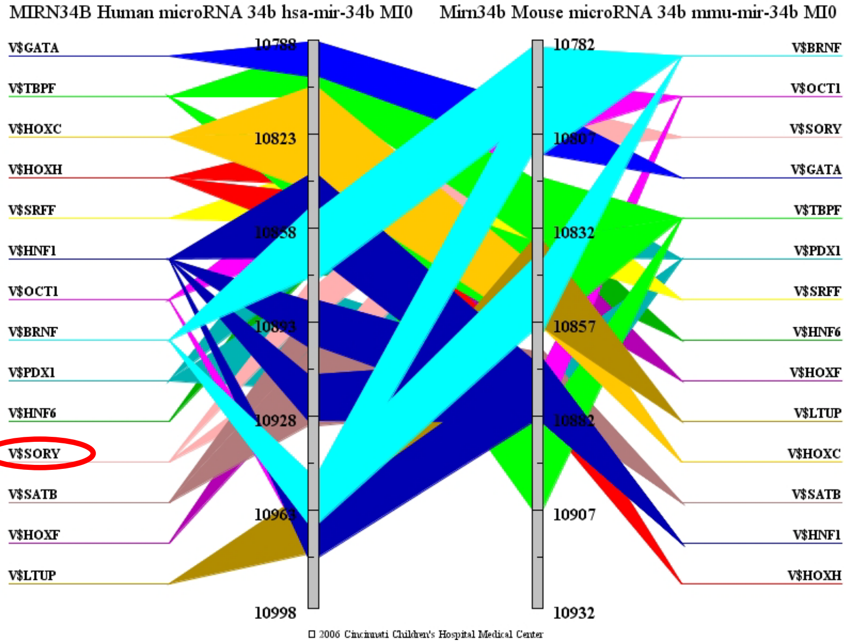

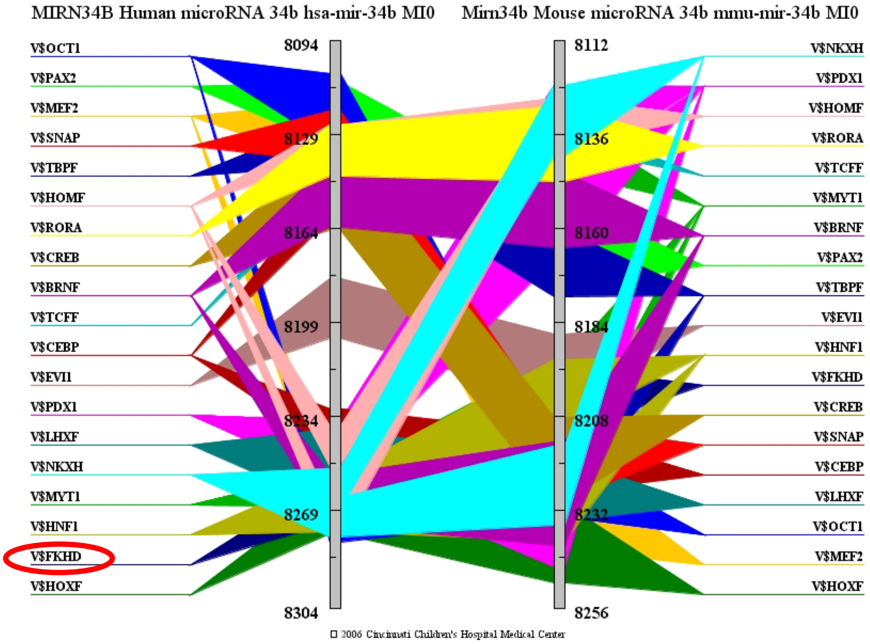

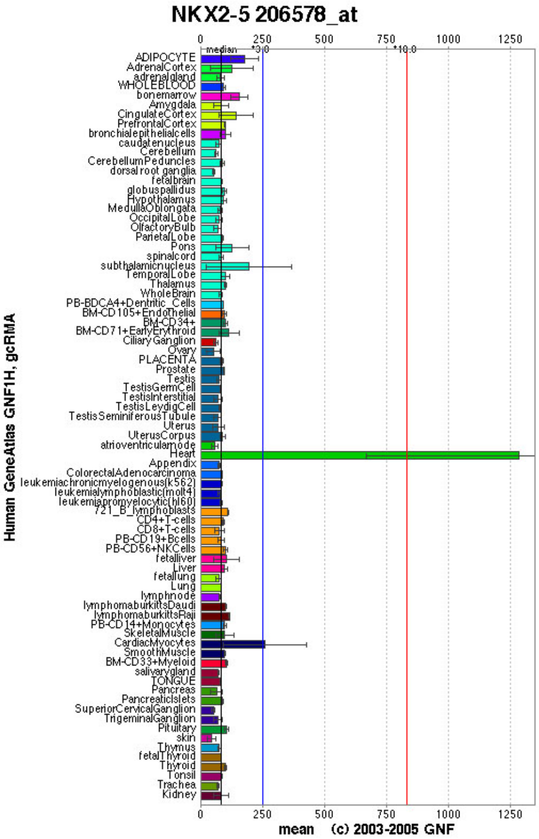

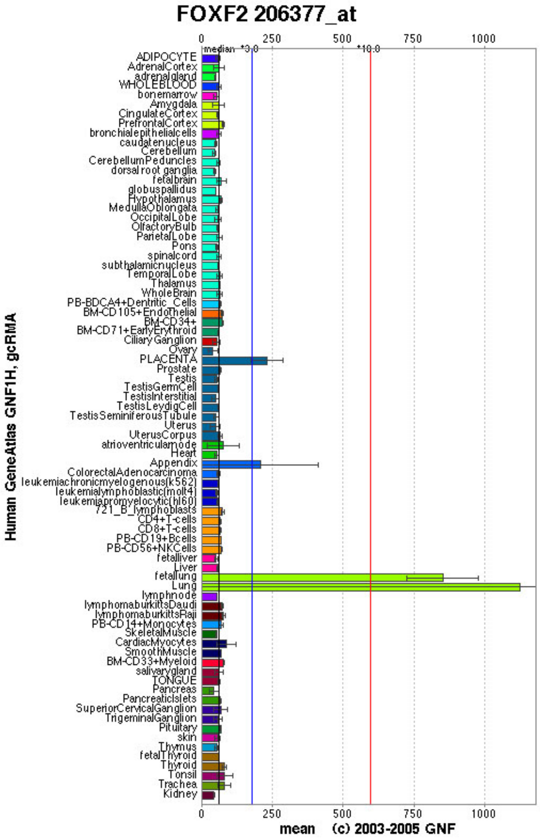

Supplement: Additional file 7 — Binding sites for Nkx2-5, SOX5, and FOXF2 and their tissue distribution. Binding sites for Nkx2-5, SOX5, and FOXF2 from GenomeTraFac, and their tissue distribution from the GNF database. [file 1471-2164-8-166-S7.pdf]
